# The safety and immunogenicity of inactivated rotavirus vaccine delivered by a dissolving microneedle patch in rats

**DOI:** 10.1080/21645515.2025.2555700

**Published:** 2025-09-11

**Authors:** Sung-Sil Moon, Marly Richter-Roche, Yuhuan Wang, Kimberly R. Foytich, Theresa K. Bessey, Cameron Feriante, Devin V. McAllister, Erin Luea, Baoming Jiang

**Affiliations:** a Division of Viral Diseases, Centers for Disease Control and Prevention (CDC), Atlanta, GA, USA; b Micron Biomedical, Inc., Atlanta, GA, USA; c Southern Research, Birmingham, AL, USA

**Keywords:** Rotavirus, inactivated rotavirus vaccine, dissolving microneedle patch, toxicology, immunogenicity

## Abstract

The development of dissolving microneedle patches (dMNPs) for inactivated rotavirus vaccine (IRV) requires concurrent evaluation of safety parameters, including local and systemic toxicity and immune response characteristics. The aim of this study is to evaluate the toxicological safety profiles and immunogenic potential of IRV dMNPs in rats through assessment of systemic toxicity, local responses, and neutralizing activities. IRV dMNPs were examined for stability under different storage conditions (5 ± 3°C and 25 ± 2°C) for up to 24 months. Toxicology assessment included clinical observation, body weight monitoring, food consumption, hematology, clinical chemistry, organ weight analysis, and histopathological examination. Local tolerance was evaluated using Draize scoring and microscopic examination of the administration sites. Immunogenicity was evaluated by measuring neutralizing activities in sera against homotypic and heterotypic RV strains. The potency of IRV dMNPs was found stable for at least 24 months at 5 ± 3°C, and for 12 months at 25 ± 2°C. Toxicological evaluation showed no adverse systemic effects, with all clinical pathology parameters remaining within normal ranges. Local dermal reactions were mild (mean Draize scores <1.5) and subsided within 48 h. Histopathology showed only low to mild inflammatory changes at the application sites, with complete resolution by the end of study (day 57). Immunological analysis showed robust and cross-reactive neutralizing activities against homotypic and heterotypic RV strains in sera of rats. IRV dMNPs showed strong stability and an excellent safety profile while also inducing robust and extensive immune responses, supporting its further development as an alternate IRV platform.

## Introduction

Rotavirus (RV) is an enteric pathogen responsible for severe diarrhea and remains a considerable global health concern, especially in low- and middle-income countries (LMICs).^1,2^ While current live-attenuated RV vaccines have shown success in reducing disease burden, their efficacy remains suboptimal in LMICs.^3,4^ Moreover, these vaccines face several limitations, including cold chain storge requirements, potential safety issues in immunocompromised individuals, and the risk of intussusception.^5,6^ To overcome these challenges, inactivated RV vaccine (IRV) has been developed as an alternative strategy and showed high immunogenicity and protection against an oral challenge with a human RV strain in animal studies.^7,8^

Currently, most pediatric vaccines are administrated by intramuscular (IM) injection, which requires trained healthcare personnel and often causes pain at the injection site. Microneedle patches (MNPs) provide an innovative, minimally invasive method for vaccine administration into the skin without using hypodermic needles.^9^ Preclinical studies have shown the efficacy of MNPs for several vaccinations, including those aimed at influenza virus, poliovirus, human papillomavirus, measles virus, hepatitis B virus and RV.^10–16^ In particular, IRV (CDC-9, G1P[8]) MNPs were found to be safe, have dose-sparing effect, and induce potent immunogenicity including mucosal immunity in animals.^17,18^

The present study investigates the toxicological profiles of IRV dMNPs in Wistar rats, focusing on systemic safety, local tolerance, and histopathological changes following repeated doses. We examined stability of IRV dMNPs at 5 ± 3°C and 25 ± 2°C for up to 24 months, assessed systemic safety by monitoring physiological parameters, analyzed local skin reactions utilizing the Draize scoring system, and performed detailed histopathological analyses of major organs and administration sites. These investigations provide crucial preclinical safety data to support the potential advancement of IRV dMNPs into clinical trials and their broader application in RV immunization strategies.

## Materials and methods

### Production of the IRV drug substance

The IRV drug substance (DS) was produced by the Walter Reed Army Institute of Research (WRAIR). CDC-9, a human G1P[8] RV strain, was cultured in Vero cells, purified and inactivated using a qualified manufacturing process to be employed for the production of vaccine bulk under GMP conditions for use in clinical trials. The DS in 50 mM HEPES, 150 mM NaCl, 5 mM CaCl_2_ [pH 7.4] supplemented with 7% D-sorbitol and 0.001% Tween 80 were inactivated at 60°C for 8 h with complete inactivation confirmed by two sequential passages and plaque assay in MA104 cells. The DS was tested for potency, residual host cell DNA and proteins, residual excipients, and endotoxin, and released according to regulatory requirements. The DS was stored at −80°C before being used to manufacture MNPs.

### MNP manufacturing

Microneedle patches (MNPs), composed of 163 microneedles, each measuring 700 µm in length, were manufactured from molds (Sylgard™ 184, Dow Corning, Midland, MI) using a two-step casting method (i.e., first an antigen-containing solution that is dried to form the MN tips followed by a polymer matrix solution that forms the lower portion of the MNs and the base from which they extend). Antigen casting solutions were prepared by mixing IRV antigens with excipients. For the fabrication of IRV dMNPs, the antigen casting solution had a concentration of 217 µg/mL IRV, 1% w/v sucrose (EMD Millipore, Burlington, MA) and 0.02% w/v methylcellulose (Spectrum Chemical Manufacturing, Gardena, CA) in 50 mM HEPES pH 7.3 (Millipore Sigma, Saint Louis, MO), 150 mM NaCl (Baxter, Deerfield, IL) and 5 mM CaCl_2_ (Hospira, Lake Forrest, IL) buffer. The polymer matrix solution used to form base of the MN arrays was composed of 27% w/w maltodextrin, 4.5% w/w sorbitol and 4.5% w/w sucrose in HEPES, NaCl, and CaCl_2_ buffer.

For dMNP fabrication, 30 µL of antigen solution was deposited into each MN array mold cavity and dried via centrifugation (30°C, 3000 xg, 2.5 h). After drying, 40 µL of polymer matrix solution was deposited, filled (centrifugation 30°C, 3000 xg, 5 min), followed by a second deposition of 85 µL of polymer matrix solution, and drying on a hot plate (45°C ramping down to 25°C, 12 h). The MN arrays were then affixed to an adhesive backing, demolded, placed in protective plastic caps, and sealed in foil pouches with desiccant. Two dMNP formulations were used; 6.5 µg IRV/patch for stability testing, and 7.1–7.3 µg IRV/patch for toxicology. In toxicology studies, two patches were applied per rat, with ~ 85% delivery efficiency, yielding a target delivered dose of ~10 µg IRV per rat.

### In vitro potency and stability study

We measured IRV potency and stability in dMNPs with Pierce™ Coomassie Bradford assay kit (Thermo Fisher Scientific, IL, USA) and a modified EIA using a RV VP7-specific monoclonal antibody (mAb).^19^ For EIA, 96-well plates were coated with rabbit anti-RV (CDC-9) polyclonal antibody overnight at 4°C. The plates were washed, blocked with Superblock™ T20 (TBS) blocking buffer (Thermo Fisher Scientific) followed by incubation with a serially diluted (two-fold) solution of reconstituted IRV dMNP samples for 1 h at 37°C. After washing, the plates were treated with biotin-conjugated anti-RV VP7 monoclonal antibody (60 ng/ml) and incubated at 37°C for 1 h. Next, diluted Pierce™ Streptavidin Poly-HRP (1:10,000) (Thermo Fisher Scientific) was added and incubated for 1 h at 37°C. The BioFX® TMB One Component HRP Microwell Substrate (Surmodics Inc., MN, USA) was added, and the reaction was stopped by 1 N HCl. The plates were read with an EIA reader (Dynex Technologies, VA, USA) at dual wavelengths of 450 nm and 630 nm. The amount of potency in the sample was determined from a curve generated using purified rotavirus standard of known concentration.

### Toxicity study in rats

#### Administration of IRV dMNPs

**Figure 1.**
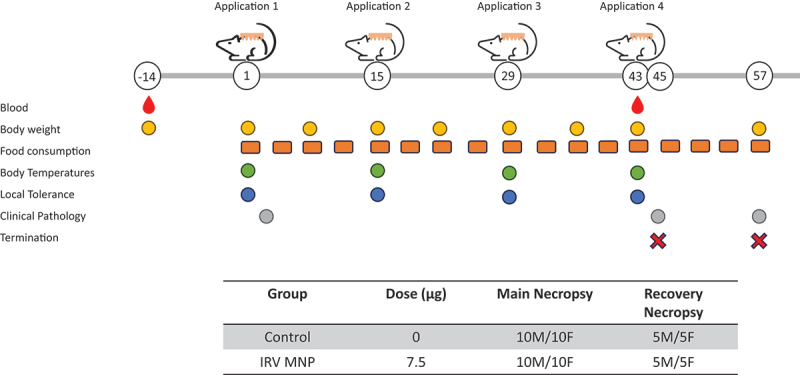
Study design and monitoring schedule for IRV MNPs safety and toxicity evaluation. The timeline shows four IRV MNPs applications (days 1, 15, 29, and 43) with assessment timepoints (−14 to 57 d). Monitoring parameters include blood sampling, body weight, food consumption, body temperature, local tolerance, clinical pathology, and termination points. Table shows group allocation, and necropsy schedule.

The non-clinical toxicology study was performed by Southern Research (Birmingham, Alabama, USA) (Figure 1). All animal experiments were approved by the Institutional Animal Care and Use Committee (IACUC) of the Southern Research and conducted in accordance with the ethical guideline for animal experiments and safety guidelines.

A total of 70 Wistar rats (35 males, 35 females, 6 weeks of age) (Charles River Laboratories, Raleigh, NC, USA) were pre-bled on day-14 (14 d prior to administration of IRV dMNPs) and screened for RV-specific IgG antibody before being selected for the study. To prepare the application site, the backs of the rats were shaved with electric shears, followed by application of a depilatory cream (Nair, NJ, USA) 1 d before dMNPs application. The dMNPs were applied to the skin, by pressing the force feedback indicator (FFI) until a click was heard, maintaining pressure for 10 s, followed by reduced force for 1 min to ensure adhesion. The dMNPs were left on the skin for 20 min, removed and placed in box. The remaining IRV in the used dMNPs was measured by EIA at CDC Rotavirus Laboratory in order to estimate the dose delivery and corresponding delivery efficiency. The repeated intradermal administration of two IRV dMNPs (3.75 µg per patch for 7.5 µg per dose of RV antigen) was performed on days 1, 15, 29, 43 in 30 RV-negative Wistar rats (15 males, 15 females). The repeated intradermal administration of placebo dMNPs was done in 30 RV-negative Wistar rats (15 males, 15 females) in the same manner. Blood samples were collected at pre-dose (Day-14) and at post dose 3 (Day 43) (Figure 1). Serum samples were stored at −80 C before being used for immunogenicity testing.

#### Clinical observation

The rats were observed at least twice daily throughout the study for signs of moribundity and mortality observations.

#### Body weights

Body weights were reordered for randomization, prior to dosing on Day 1, on Day 2, then once weekly throughout the study. The final body weights were collected prior to necropsy. Food consumption was measured for all animals once weekly beginning during the week prior to dose initiations.

#### Food consumption

Food consumption was assessed for all animals three or four times weekly, starting the week before the initiation of each dose.

#### Body temperature

Body temperature was measured in all animals by implanted microchip on Day 1, 15, 29 and 43 (prior to dosing and post dosing at 6 h ±10 min and 24 h ±20 min).

#### Local tolerance

The administration site was evaluated for local tolerance on Day 1, 15, 29, and 43 (prior to and post administration at approximately 1, 4, 14, 48, and 72 h) (Figure 1). The administration sites were individually graded using the modified Draize score (erythema/edema) (supplementary table S1) and other reactions (e.g., rash, oozing, hair loss, crusting, and ulceration) were recorded as noted.

#### Clinical pathology

The clinical pathology samples were collected from all animals prior to dose administration and on Day 3, 45, and 57. The hematology samples, approximately 0.5 mL, were collected in tubes containing EDTA anticoagulant (Suplementary Table S2). The measured parameters included red blood cell count (RBC), white blood cell count (WBC), mean corpuscular hemoglobin (MCH), hemoglobin concentration (HGB), platelet count (PLT), hematocrit (HCT), mean corpuscular volume (MCV), and differential leukocyte counts. The clinical chemistry samples were collected in tubes without anticoagulant (Suplementary Table S3). After centrifugation, samples were analyzed for the following parameters: alanine aminotransferase (ALT), aspartate aminotransferase (AST), alkaline phosphatase (ALP), gamma-glutamyl transferase (GGT), total bilirubin (TBIL), blood urea nitrogen (BUN), chloride (Cl), phosphorus (Phos), total protein (TP), albumin, globulin (Alb), albumin/globulin ratio (A/G ratio), and glucose (Gluc). Coagulation samples were collected in tubes containing sodium citrate anticoagulant and assayed for prothrombin time, partial thromboplastin time, and fibrinogen.

### Pathology

#### Macroscopic pathology

All animals had a comprehensive postmortem examination. The postmortem examination encompassed the assessment of the body’s external surfaces, as well as the cranial, thoracic, abdominal, and pelvic cavities and their contents. Tissues were procured for microscopic pathology and assessed as detailed below: adrenal gland, brain, epididymis, eye, heart, kidney, large intestine (cecum), large intestine (colon), liver, lungs, lymph node, ovary with oviduct, parathyroid, pituitary, prostate, salivary gland, seminal vesicle, skin (administration site), small intestine (duodenum, ileum, jejunum), spleen, stomach, testis, thymus, thyroid, urinary bladder, and uterus. The following organs were weighed, with paired organs weighed together and recorded as a combined weight, brain, heart, kidney, liver, lungs, ovary with oviduct, pituitary, prostate, seminal vesicle, spleen, testis, thymus, and thyroid, parathyroid, and uterus.

#### Microscopic pathology

The identification of each animal was maintained through the tissues collected during necropsy. All tissues and organs, except for the testes, epididymis, and eyes, were preserved in 10% neutral-buffered formalin. Eyes were preserved in Davidson’s solution, whereas the testes and epididymis were preserved in modified Davidson’s solution. Residual carcasses were disposed of after necropsy. Tissues obtained from all animals were preserved for histopathological analysis. Fixed tissues, along with the aforementioned and any gross lesions noted in all animals across all groups and time points, were trimmed, processed, and sectioned into approximately 5-micron slices. Tissue sections were affixed to glass slides, subjected to hematoxylin and eosin staining, and subsequently covered for microscopic analysis.

#### Immunogenicity study

RV-specific IgG in sera at −14 d of administration was measured using a modified enzyme immunoassay^16^ to identify RV-free animals. RV-specific neutralizing activity (NA) in sera was measured with a microneutralization assay against homotypic RV strain, Wa (G1P[8])^20^ and heterotypic RV strain CDC-6 (G9P[6]).^16^ Each RV strain was individually optimized to use 700 FFU for Wa and 2,400 FFU for CDC-6 per well. Neutralizing titer was defined as the reciprocal of the highest dilution that gave a greater than 70% reduction in the absorbance value compared to that in RV only control wells.

#### Statistical analysis

A comparative statistical analysis of body weight, food consumption, organ weights, and clinical pathology data was conducted using Provantis®. The Shapiro–Wilk test was employed to assess normality, whereas the Levene test was utilized to establish homogeneity of variance. A non-parametric one-way ANOVA was conducted based on the test results, followed by an appropriate post-hoc pairwise analysis. The significance level was *p* < .05 (*p* < .01 for normality and variance assessments). All immunogenicity results were analyzed by Graphpad Prism software version 7 (GraphPad, CA, USA). Comparisons among individual samples were done using an unpaired t test. Comparisons among multiple groups were done using a two-way ANOVA (*p* < .05 was considered significant).

## Results

### Stability of IRV dMNP

**Figure 2.**
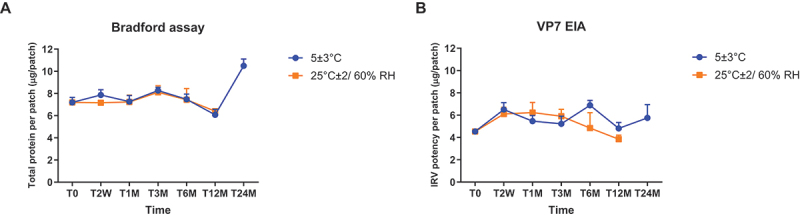
Stability assessment of IRV MNP by Bradford assay and VP7 EIA under different storage conditions. (A) the Bradford assay measuring total protein concentration over 24 months at 5 ± 3°C (blue and 25°C ± 2/60% RH (orange). Error bars represent standard deviation. (B) the VP7-specific EIA results showed antigen stability over 24 months under the same storage condition.

IRV dMNPs were stored (5 ± 3°C or 25 ± 2°C/60% relative humidity) and stability tested for up to 24 months (Figure 2). The dMNPs had a nominal dose of 6.5 µg of total protein, determined by Bradford assay. All IRV dMNPs at 5 ± 3°C storage maintained expected potency and thus were stable for at least 24 months. The dMNPs stored at 25 ± 2°C maintained stability for at least 12 months, though the concentration showed a slight decrease at 12 months. Similar stability data were observed by RV EIA. The IRV dMNPs were monitored for appearance; all appeared clear to slightly off-white, with correct physical form, and passed appearance tests up to 24 months.

T0-T24M indicate sampling timepoints (T0: initial, W: weeks, M: months).

### Manufacturing consistency and delivery efficiency study in rats

IRV contents, as measured by the Bradford assay, ranged from 7.1 to 7.3 µg/patch for four batches produced to support the GLP-toxicology study. The delivery efficiency and dose delivered of IRV MNPs were evaluated by VP7 EIA across all animals and four application timepoints using three separate manufacturing batches (Table 1). The overall delivery efficiency across four applications was 73%, with a given dose of approximately 10.5 µg per rat.

**Table 1.** Delivery efficiency and dose distribution of protein dosage across applications and batches in inactivated rotavirus vaccine microneedle patches (IRV MNPs).

Application	Batch #	Protein content^a^ (µg/patch)	Delivery efficiency^b^ (%)	Average dose delivered/patch	Average dose delivered/rat
1	1	7.2	69%	5.0	10
2	2	7.3	69%	5.0	10
3	3	7.1	69%	4.9	9.8
4	2 and 3	7.2	85%	6.1	12.2

^a^Protein content was measured by the Bradford assay.

^b^Delivery efficiency was estimated [((initial dose−residual dose)/initial dose) × 100%] by measuring IRV content of the dMNPs before and after administration by RV VP7 EIA.

## Toxicology study

### Clinical observations

No animals in distress were seen during the bi-daily cage-side mortality and morbidity assessments throughout the study. Clinical findings were normal in all animals within both placebo and IRV dMNPs groups. Observation abnormalities were confined to sporadic scabs or discoloration on several rats, primarily attributable to routing grooming and behavior rather than from the treatment. The administration of the IRV dMNPs was well tolerated across placebo and IRV dose levels.

### Body weights

**Figure 3.**
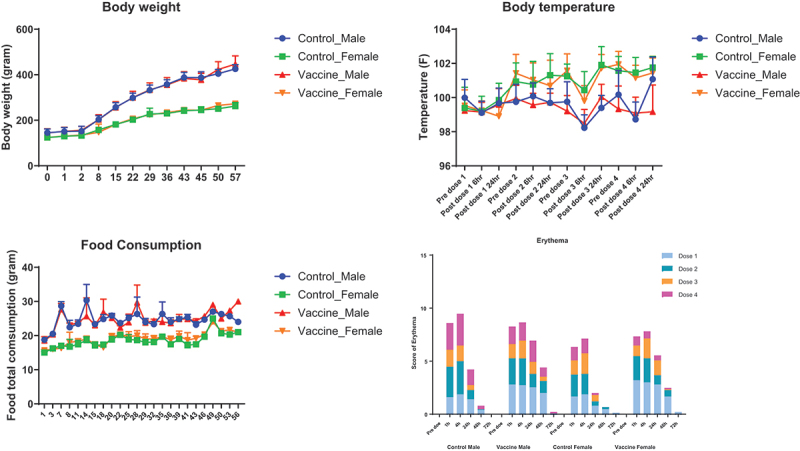
Clinical observation parameters following IRV MNP administration. Body weight, food consumption, and body temperature were monitored by sex and treatment group (placebo and IRV MNP) throughout the study. Erythema score at application sites were assessed for each dose, showing temporal changes in local reactions across placebo and IRV MNPs groups. *symbols indicate statistically significant differences between groups: *p* < .05.

Longitudinal body weights showed that during the dosing and recovery phases, the growth patterns of both male and female animals in the IRV dMNPs group cohorts were similar to those in the placebo groups (Figure 3(A)). The IRV dMNPs showed no effect on body weight. Male animals in both placebo and IRV dMNPs groups consistently showed greater body weight gain than females, indicating typical sexual dimorphism.

### Food consumption

The data on food consumption did not clearly show any trends in intake between placebo and IRV dMNPs groups, regardless of sex (Figure 3(B)). Male animals in both placebo and IRV dMNPs groups showed variability in their consumption pattern. Both the female placebo and IRV dMNPs showed little variation, which suggests a consistent, although diminished, consumption pattern. IRV administration by dMNPs did not cause any dose-related effect on food consumption.

### Body temperatures

Compared to the placebo animals, there were no significant changes in IRV dMNPs group temperatures in male animals during the study (Figure 3(C)). Several individual animals had slightly elevated body temperature in both the placebo and IRV dMNPs dose groups; however, the changes were highly variable and not considered adverse.

Starting on Day 15, all females in the placebo and IRV dMNPs group showed elevated body temperatures during all collection intervals. The increased body temperatures seen in both groups of females during pre- and post-dose collections were not deemed directly related to vaccine dose administration and were not considered adverse effects.

### Local tolerance

Local tolerance evaluations using the Draize scoring system were conducted on a 5-point scale for both erythema and edema (Figure 3(D)). Erythema grades ranging from 0 to 2 were recorded at all time points for both male and female animals in the placebo and IRV dMNPs groups. Most instances of irritation at the administration site resolved in 48 h, with all erythema clearing by 72 h following dMNPs administration. A slight incidence of edema was noted in male animals from the placebo group an hour after the second dose on Day 15, which resolved within 4 h of administration (data not shown). The low scores, which resolved spontaneously in both the placebo and IRV dMNPs groups, indicated that the administration site reactions were not considered detrimental. Overall, the IRV dMNPs group showed no sign of edema.

### Clinical pathology

We examined hematological parameters on samples from a specific subset of animals on Day 3 and from all animals on their designated necropsy days at Day 45 or 57 (Table 2). On Day 45 and 57, the IRV dMNPs group animals showed a significant increase in white blood cell (WBC) counts. Furthermore, lymphocyte and eosinophil counts were increased in both IRV dMNPs administered males and females compared to the placebo, although monocyte counts were exclusively increased in IRV dMNPs females.

**Table 2.** Hematology and chemistry parameters in male and female subjects treated with inactivated rotavirus vaccine microneedle patches (IRV dMnps) or placebo dMnps.

Parameter	Gender	Group	Day 3	Day 45	Day 57
**Hematology**					
WBC (10³/mL)	Male	Placebo	7.97 ± 1.39	8.53 ± 1.51	7.11 ± 1.76
		Vaccine	7.62 ± 1.53	10.01 ± 2.78	8.01 ± 2.28
	Female	Placebo	7.10 ± 2.09	6.70 ± 2.58	4.93 ± 1.20
		Vaccine	7.80 ± 2.19	5.49 ± 1.25	5.63 ± 2.61
Lymph (10³/mL)	Male	Placebo	6.16 ± 1.17	6.24 ± 1.60	5.59 ± 1.50
		Vaccine	5.55 ± 1.36	7.98 ± 2.43	5.87 ± 1.48
	Female	Placebo	5.10 ± 1.51	4.54 ± 1.61	3.68 ± 1.08
		Vaccine	5.61 ± 1.90	3.86 ± 0.96	4.45 ± 2.41
Eosinophil (10³/mL)	Male	Placebo	0.10 ± 0.03	0.28 ± 0.09	0.14 ± 0.04
		Vaccine	0.09 ± 0.03	0.22 ± 0.14	0.36 ± 0.36
	Female	Placebo	0.12 ± 0.07	0.33 ± 0.23	0.16 ± 0.03
		Vaccine	0.14 ± 0.07	0.22 ± 0.14	0.13 ± 0.06
**Chemistry Parameters**					
BUN (mg/dL)	Male	Control	12.22 ± 1.58	18.80 ± 1.96	17.78 ± 1.08
		Vaccine	11.72 ± 1.77	19.31 ± 2.21	20.32 ± 1.63
	Female	Control	12.85 ± 2.88	18.90 ± 2.57	20.52 ± 2.19
		Vaccine	13.16 ± 2.70	21.70 ± 2.17	19.84 ± 3.37
Glucose (mg/dL)	Male	Control	170.60 ± 8.41	175.40 ± 24.54	189.20 ± 19.21
		Vaccine	165.20 ± 10.66	166.80 ± 17.55	164.60 ± 19.18
	Female	Control	169.75 ± 15.82	162.20 ± 13.84	145.20 ± 23.49
		Vaccine	155.60 ± 12.36	138.90 ± 20.07	142.80 ± 15.07
Phos (mg/dL)	Male	Control	7.48 ± 0.20	6.43 ± 0.66	6.42 ± 0.50
		Vaccine	7.94 ± 0.17	7.09 ± 0.50	6.60 ± 0.59
	Female	Control	7.23 ± 0.74	5.10 ± 0.65	5.88 ± 0.40
		Vaccine	7.84 ± 0.19	6.73 ± 1.11	5.94 ± 0.50
AST (U/L)	Male	Control	134.80 ± 41.19	88.40 ± 11.61	84.20 ± 5.07
		Vaccine	142.75 ± 26.41	90.20 ± 15.82	102.00 ± 16.51
	Female	Control	105.75 ± 10.21	78.00 ± 11.33	85.00 ± 11.38
		Vaccine	145.80 ± 24.11	101.60 ± 18.96	87.20 ± 14.34
K (mmol)	Male	Control	5.78 ± 0.36	4.76 ± 0.25	4.84 ± 0.09
		Vaccine	5.58 ± 0.11	5.02 ± 0.31	5.06 ± 0.27
	Female	Control	5.14 ± 0.18	4.21 ± 0.21	4.26 ± 0.30
		Vaccine	5.40 ± 0.39	4.70 ± 0.34	4.36 ± 0.18
Cholesterol (mg/dL)	Male	Control	116.40 ± 12.36	98.50 ± 17.13	78.80 ± 13.14
		Vaccine	108.80 ± 10.64	98.10 ± 19.09	82.40 ± 8.32
	Female	Control	108.20 ± 13.77	92.30 ± 5.06	99.00 ± 16.54
		Vaccine	123.00 ± 4.06	97.10 ± 9.17	111.80 ± 5.81

The levels of blood urea nitrogen (BUN) slightly increased in male IRV dMNPs groups on Day 45 and followed by a significant increase (14.3%) on Day 57. On Day 45, phosphorus levels increased by 10.3% compared to the placebo group; however, the percentage change decreased to 2.8% by Day 57. Glucose levels consistently showed reductions compared to the placebo group in all study periods, with a range of 3.2% to 13.0% lower values. In IRV dMNPs, females showed increased aspartate transferase (AST) levels on Day 3 and Day 34, with increases of 37.8% and 30.3%, respectively, which are statistically significant. These values decreased following the recovery period. Additionally, BUN levels were increased on Day 3 (13.6%) and Day 45 (14.8%) as compared to the placebo group females. IRV dMNPs females also showed increased potassium and phosphorus on Day 45, with a recovery by Day 57. When compared to females in placebo group, glucose decreased on all days, with a percent change ranging from 8.3% to 14.4% on Day 3 and 45. By Day 57, only a 1.7% change was present. Alternatively, there was an increase in cholesterol on all study days, with a percentage increase ranging from 5.2% to 13.7% compared to the females in the placebo group.

All coagulation data showed inconsistent changes, and neither the males nor females in the IRV dMNPs or placebo groups showed a consistent trend of increase or reduction.

### Pathology

During the Day 45 necropsy, crusts were noted at the dosage administration site of 4 out of 10 females in the IRV dMNPs group. Furthermore, malformation of the right cerebrum, accumulation inside the cranial cavity, mottled lung discoloration, enlargement of the mandibular lymph nodes, darkening of the mediastinal lymph node, and uterine dilation were also noted. None of these lesions were observed on the Day 57 necropsy evaluations.

On Days 45 and 57, the thymus-to-brain weight ratio for IRV dMNP females was statistically significantly higher than that of placebos, at 56% and 18%, respectively. The differences in organ weight did not correspond to microscopic observations and were also considered incidental.

Microscopic examination on Day 45 showed that applying dMNP was associated with active inflammation of the skin. The severity of inflammation was slightly greater in animals administrated IRV dMNP compared to those given a placebo controls, with females exhibiting marginally greater severity than males. The inflammation was observed in 30% (3 animals) of males in the placebo group and 100% of males in IRV dMNPs group and females in both IRV dMNPs and placebo groups. Most inflammation was minimal to mild, with moderate inflammation note in only one animal (10%) of IRV dMNPs females. The inflammation was reduced in all groups by Day 57.

The IRV dMNPs appeared to cause mild-to-moderate skin inflammation and ulceration, with severity comparable to placebo groups. These transient local reactions within 2 weeks, supporting an improved safety profile.

### Immunogenicity

**Figure 4.**
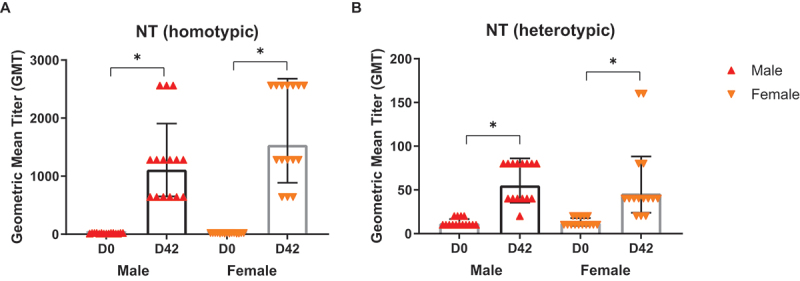
Neutralizing antibody responses against homotypic and heterotypic RV strains. Geometric mean titer (GMT) measured at baseline (D0) and day 42 (D42) post-IRV MNPs administration separated by sex. Bars represent 95% confidence intervals.

The immunogenicity of IRV dMNPs was evaluated by measuring neutralizing activity against homotypic and heterotypic RV strains in sera of vaccinated rats (Figure 4). For homotypic neutralization, no geometric mean titers (GMT) at baseline (D0) (<10 GMT) were detected in both male and female rats (Figure 4(A)). These results were consistent with the absence of detectable IgG levels at Day −14 prior to dMNP administration (data not shown). After the third application, substantial increases in NT were observed at D42 (*p* < .05). Male rats showed a GMT of approximately 1,000, with individual responses ranging from 600 to 2,500. Female rats showed slightly higher responses, achieving a GMT of 1,500, with individual values also ranging from 600 to 2,500. The data showed some variability between individual animals, as indicated by the error bars. Heterotypic NT using a single G9P[6] strain (CDC-6) showed modest but measurable cross-reactive responses (*p* < .05) (Figure 4(B)). By D42, males developed a GMT of approximately 50, with individual responses ranging from 30 to 80. Similarly, females reached a GMT of 45, though with a wider individual variation (20–160). The fact that heterotypic NT was present suggests that vaccination with a G1P[8] IRV dMNP could provide cross-protection against heterologous RV strains. The similar responses from males and females suggest that sex does not significantly influence the vaccine’s ability to induce neutralizing antibodies.

## Discussion

This study showed that four doses of intradermally administered IRV using a dMNP caused no morbidity and mortality, no adverse clinical observations in rats, and thus established the proof of concept for the safety of this product. These safety and robust immunogenicity data in rats, together with published studies that demonstrated antigen dose-sparing effect and intestinal immunity,^17^ support clinical testing of IRV dMNPs in humans.

We demonstrated consistent protein content and potency across batches by both Bradford test and EIA, thus ensuring reliable manufacturing quality. The delivery efficiency was 70 ~ 85% for individual batches, indicating robust functioning performance and precise doses of the IRV MNP.

Our toxicology study showed that there were no significant systemic adverse effects in rats, with slightly mild, transient local reactions that resolved within 48 h. These findings provide critical evidence that the vaccine does not induce systemic effects significant enough to alter growth rates, an important safety consideration in preclinical studies. Body weight is often a sensitive marker of toxicity, and the lack of adverse effects on this parameter suggests that the vaccine formulation, as delivered by the dMNP, was well tolerated in animals.

The present study demonstrated that IRV MNP induced neutralizing activities against both homotypic and heterotypic RV strains, confirming the previous studies in animal models.^16–18, 21^ The cross-protective responses are particularly noteworthy given the global diversity of circulating RV genotypes and the current limitation of live-attenuated RV vaccinations in LMICs.^22^ The potential of IRV dMNPs to enhance both systemic and mucosal immunity while providing dose-sparing benefits represents a significant progression in the development of RV vaccines.

We showed that IRV dMNPs maintained potency at both refrigerated (5 ± 3°C) and long-term (25 ± 2°C/60% RH) storage conditions for up to 24 months. This stability profile exceeded that of live-attenuated vaccines and would overcome a major challenge in global immunization program, the requirement for large-scale cold-chain storage. Our findings corroborated previous studies on MNP thermostability^11^ and indicate that the dMNP platform could significantly improve vaccination accessibility in regions with limited cold-chain infrastructure.

Although the anatomical discrepancies between rat and human skin are acknowledged constraints of animal studies, previous clinical trials offer compelling proof for the effective and safe use dissolvable microneedle patches in humans. A phase 1 randomized clinical trial revealed that a dissolvable microneedle patch administering inactivated influenza vaccine was well tolerated, immunogenic, and favorably received by adult participants.^23,24^ These findings, with current clinical study of the microneedle-delivered rotavirus vaccine, support the potential applicability of this vaccine delivery system to human populations and mitigate concerns related to species differences in skin.

In conclusion, this GLP-enabling toxicology study provides strong proof of concept for the safety, immunogenicity, and stability characteristics of IRV MNP in rats. Studies are under way in testing safety and immunogenicity of this novel vaccine in a phase 1 clinical trial in healthy adults.

## Supplementary Material

Supplement.docx

## Data Availability

The data that support the findings of this study are available from the corresponding author upon reasonable request.

## References

[1] Tate JE, Burton AH, Boschi-Pinto C, Parashar UD, World Health Organization-Coordinated Global Rotavirus Surveillance N. Global, regional, and national estimates of rotavirus mortality in children <5 years of age, 2000–2013. Clin Infect Dis. 2016;62(Suppl 2):S96–12. doi: 10.1093/cid/civ1013. 27059362 PMC11979873

[2] WHO. Rotavirus vaccines: WHO position paper - July 2021. 2021: 301–319.

[3] Jiang V, Jiang B, Tate J, Parashar UD, Patel MM. Performance of rotavirus vaccines in developed and developing countries. Hum Vaccin. 2010;6(7):532–542. doi: 10.4161/hv.6.7.11278. 20622508 PMC3322519

[4] Church JA, Parker EP, Kirkpatrick BD, Grassly NC, Prendergast AJ. Interventions to improve oral vaccine performance: a systematic review and meta-analysis. Lancet Infect Dis. 2019;19(2):203–214. doi: 10.1016/S1473-3099(18)30602-9. 30712836 PMC6353819

[5] Tate JE, Yen C, Steiner CA, Cortese MM, Parashar UD. Intussusception rates before and after the introduction of rotavirus vaccine. Pediatrics. 2016;138(3). doi: 10.1542/peds.2016-1082. PMC11973882 27558938

[6] Chiu M, Bao C, Sadarangani M. Dilemmas with rotavirus vaccine: the neonate and immunocompromised. Pediatr Infect Dis J. 2019;38(6S):SS43–SS46. doi: 10.1097/INF.0000000000002322. 31205244

[7] Wang Y, Azevedo M, Saif LJ, Gentsch JR, Glass RI, Jiang B. Inactivated rotavirus vaccine induces protective immunity in gnotobiotic piglets. Vaccine. 2010;28(33):5432–5436. doi: 10.1016/j.vaccine.2010.06.006. 20558244

[8] Jiang B, Wang Y, Glass RI. Does a monovalent inactivated human rotavirus vaccine induce heterotypic immunity? Evidence from animal studies. Hum Vaccin Immunother. 2013;9(8):1634–1637. doi: 10.4161/hv.24958. 23744507 PMC3906259

[9] Kim YC, Park JH, Prausnitz MR. Microneedles for drug and vaccine delivery. Adv Drug Deliv Rev. 2012;64(14):1547–1568. doi: 10.1016/j.addr.2012.04.005. 22575858 PMC3419303

[10] Sullivan SP, Koutsonanos DG, Del Pilar Martin M, Lee JW, Zarnitsyn V, Choi SO, Murthy N, Compans RW, Skountzou I, Prausnitz MR. Dissolving polymer microneedle patches for influenza vaccination. Nat Med. 2010;16(8):915–920. doi: 10.1038/nm.2182. 20639891 PMC2917494

[11] Kolluru C, Gomaa Y, Prausnitz MR. Development of a thermostable microneedle patch for polio vaccination. Drug Deliv Transl Res. 2019;9(1):192–203. doi: 10.1007/s13346-018-00608-9. 30542944 PMC6328527

[12] Corbett AJ, Caminschi I, McKenzie BS, Brady JL, Wright MD, Mottram PL, Hogarth P, Hodder A, Zhan Y, Tarlinton D, et al. Antigen delivery via two molecules on the CD8- dendritic cell subset induces humoral immunity in the absence of conventional “danger”. Eur J Immunol. 2005;35(10):2815–2825. doi: 10.1002/eji.200526100. 16143986

[13] Choi Y, Lee GS, Li S, Lee JW, Mixson-Hayden T, Woo J, Xia D, Prausnitz MR, Kamili S, Purdy MA, et al. Hepatitis B vaccine delivered by microneedle patch: immunogenicity in mice and rhesus macaques. Vaccine. 2023;41(24):3663–3672. doi: 10.1016/j.vaccine.2023.05.005. 37179166 PMC10961677

[14] Edens C, Collins ML, Ayers J, Rota PA, Prausnitz MR. Measles vaccination using a microneedle patch. Vaccine. 2013;31(34):3403–3409. doi: 10.1016/j.vaccine.2012.09.062. 23044406 PMC3706567

[15] Pattani A, McKay PF, Garland MJ, Curran RM, Migalska K, Cassidy CM, Malcolm RK, Shattock RJ, McCarthy HO, Donnelly RF. Microneedle mediated intradermal delivery of adjuvanted recombinant HIV-1 CN54gp140 effectively primes mucosal boost inoculations. J Cont Release. 2012;162(3):529–537. doi: 10.1016/j.jconrel.2012.07.039. PMC3778941 22960496

[16] Moon SS, Richter-Roche M, Resch TK, Wang Y, Foytich KR, Wang H, Mainou BA, Pewin W, Lee J, Henry S, et al. Microneedle patch as a new platform to effectively deliver inactivated polio vaccine and inactivated rotavirus vaccine. NPJ Vaccin. 2022;7(1):26. doi: 10.1038/s41541-022-00443-7. PMC8885742 35228554

[17] Moon S, Wang Y, Edens C, Gentsch JR, Prausnitz MR, Jiang B. Dose sparing and enhanced immunogenicity of inactivated rotavirus vaccine administered by skin vaccination using a microneedle patch. Vaccine. 2013;31(34):3396–3402. doi: 10.1016/j.vaccine.2012.11.027. 23174199 PMC4610374

[18] Resch TK, Wang Y, Moon SS, Joyce J, Li S, Prausnitz M, Jiang B. Inactivated rotavirus vaccine by parenteral administration induces mucosal immunity in mice. Sci Rep. 2018;8(1):561. doi: 10.1038/s41598-017-18973-9. 29330512 PMC5766576

[19] Moon SS, Wang H, Brown K, Wang Y, Bessy T, Greenberg HB, Jiang B. Development and validation of a VP7-specific EIA for determining the potency and stability of inactivated rotavirus vaccine. J Virol Methods. 2024;332:115079. doi: 10.1016/j.jviromet.2024.115079. 39608463

[20] Jiang B, Estes MK, Barone C, Barniak V, O’Neal CM, Ottaiano A, Madore HP, Conner ME. Heterotypic protection from rotavirus infection in mice vaccinated with virus-like particles. Vaccine. 1999;17(7–8):1005–1013. doi: 10.1016/S0264-410X(98)00317-X. 10067709

[21] Wang Y, Vlasova A, Velasquez DE, Saif LJ, Kandasamy S, Kochba E, Levin Y, Jiang B. Skin vaccination against rotavirus using microneedles: proof of concept in gnotobiotic piglets. PLOS ONE. 2016;11(11):e0166038. doi: 10.1371/journal.pone.0166038. 27824918 PMC5100943

[22] Velasquez DE, Wang Y, Jiang B. Inactivated human rotavirus vaccine induces heterotypic antibody response: correction and development of IgG avidity assay. Hum Vaccin Immunother. 2015;11(2):531–533. doi: 10.4161/21645515.2014.988553. 25692974 PMC4514286

[23] Rouphael NG, Paine M, Mosley R, Henry S, McAllister DV, Kalluri H, Pewin W, Frew PM, Yu T, Thornburg NJ, et al. The safety, immunogenicity, and acceptability of inactivated influenza vaccine delivered by microneedle patch (TIV-MNP 2015): a randomised, partly blinded, placebo-controlled, phase 1 trial. Lancet. 2017;390(10095):649–658. doi: 10.1016/S0140-6736(17)30575-5. 28666680 PMC5578828

[24] Adigweme I, Yisa M, Ooko M, Akpalu E, Bruce A, Donkor S, Jarju LB, Danso B, Mendy A, Jeffries D, et al. A measles and rubella vaccine microneedle patch in the Gambia: a phase 1/2, double-blind, double-dummy, randomised, active-controlled, age de-escalation trial. Lancet. 2024;403(10439):1879–1892. doi: 10.1016/S0140-6736(24)00532-4. 38697170 PMC11099471

