# Supplementary material for: The safety and immunogenicity of inactivated rotavirus vaccine delivered by a dissolving microneedle patch in rats
Source: Hum Vaccin Immunother. 2025 Sep 11;21(1):2555700. doi: 10.1080/21645515.2025.2555700 (PMC12439544; doi:10.1080/21645515.2025.2555700)
Supplement: Supplement.docx [file KHVI_A_2555700_SM9950.docx]

**Table 1. Draize local irritation scoring system**

| **Draize Local Irritation Scoring System** |  |
| --- | --- |
| **Reaction - Erythema and Eschar Formation** | **Score** |
| No erythema | 0 |
| Very slight erythema (barely perceptible) | 1 |
| Well-defined erythema | 2 |
| Moderate to severe erythema | 3 |
| Severe erythema (beet redness) to slight eschar formation (injuries in depth) | 4 |
| **Reaction - Edema** | **Score** |
| No edema | 0 |
| Very slight edema (barely perceptible) | 1 |
| Slight edema (edges of area well-defined by definite raising) | 2 |
| Moderate edema (raised approximately 1 mm) | 3 |
| Severe edema (raised more than 1 mm and extending beyond area of exposure) | 4 |

**Table 2. Hematology Parameters**

| Red blood cell count  Hemoglobin concentration  Hematocrit  Mean corpuscular volume  Mean corpuscular hemoglobin concentration | Mean corpuscular hemoglobin  Platelet count  White blood cell count  Differential Leukocyte counts  (Relative and Absolute) |
| --- | --- |

## Table 3. Clinical Chemistry Parameters

| Alanine aminotransferase  Aspartate aminotransferase  Alkaline phosphatase  Gamma-glutamyl transferase  Total bilirubin Urea nitrogen  Creatinine  Sodium  Potassium  Calcium | Chloride  Phosphorus  Total Protein  Albumin  Globulin  Albumin/globulin ratio  Glucose  Cholesterol  Triglycerides |
| --- | --- |
